# Predictive value of α-synuclein expression in peripheral blood of multiple sclerosis patients: A two-dimensional assessment of a selected biomarker

**DOI:** 10.1371/journal.pone.0285022

**Published:** 2023-08-03

**Authors:** Rabat Razia, Fazeel Majeed, Rehab Amin, Shahid Mukhtar, Khalid Mahmood, Turki Abualait, Shahid Bashir, Deeba Noreen Baig

**Affiliations:** 1 School of Life Sciences, Forman Christian College (A Chartered University) Lahore, Lahore, Pakistan; 2 Rashid Latif Medical College, Lahore, Pakistan; 3 Punjab Institute of Neurosciences, Lahore, Punjab, Pakistan; 4 College of Applied Medical Sciences, Imam Abdulrahman Bin Faisal University, Dammam, Saudi Arabia; 5 Neuroscience Center, King Fahad Specialist Hospital, Dammam, Saudi Arabia; University of Campania Luigi Vanvitelli: Universita degli Studi della Campania Luigi Vanvitelli, ITALY

## Abstract

**Introduction:**

Our study aimed to evaluate whether assessing α-synuclein expression levels in blood samples could provide a reliable and straightforward alternative to existing diagnostic and prognostic methods for neurodegenerative disorders, including multiple sclerosis (MS). We specifically investigated if α-synuclein and IL-6 expression levels from serum and peripheral blood mononuclear cells (PBMCs) could accurately predict MS severity in patients using a two-dimensional approach.

**Methods:**

We designed a case-control study to analyze the expression of α-synuclein and IL-6 in the peripheral blood of an MS patient group (n = 51) and a control group (n = 51). We statistically evaluated the PBMCs and serum profiles of α-synuclein and IL-6 in MS patients, along with their age of onset, disease duration, tobacco exposure, and Expanded Disability Status Scale (EDSS) score, using SPSS V22.0 software and GraphPad Prism V9.0.

**Results:**

Our findings indicate that α-synuclein production was significantly downregulated in MS patients. Principal component analysis also revealed distinct profiles between MS patients and controls. PBMCs and serum profiles of α-synuclein correlated with the EDSS score, suggesting that disease severity can be predicted using α-synuclein profiles. Moreover, α-synuclein showed a significant correlation with IL-6 and age of onset. Lastly, receiver operating characteristic curves of PBMCs and serum activity of α-synuclein profiles displayed discrimination with area under the curve values of 0.856 and 0.705, respectively.

**Conclusion:**

Our results imply that measuring α-synuclein levels in both serum and PBMCs could be a valuable method for diagnosing and predicting MS severity, potentially serving as a non-invasive biomarker for the disease.

## 1. Introduction

Multiple sclerosis (MS) is an unpredictable autoimmune neurodegenerative disease affecting the central nervous system, characterized by demyelination, the significant loss of the myelin sheath, which results in the formation of plaques in the brain and spinal cord [1]. MS is the primary cause of disability among young adults and is twice as common in women compared to men [2]. The four subtypes of MS–relapsing-remitting MS (RRMS), progressive RRMS (PRMS), primary progressive MS (PPMS), and secondary progressive MS (SPMS)–differ in prevalence, with RRMS accounting for 80–87% of all MS cases [3]. Approximately 10–15% of patients are initially diagnosed with PPMS, and nearly 65% of those diagnosed with RRMS eventually develop SPMS, classified as the second MS stage [4]. Another MS category, clinically isolated syndrome (CIS), refers to the first episode of MS resulting from demyelination [5].

Although the etiology of MS remains unclear, it is widely accepted that immune dysregulation triggered by genetic and environmental factors causes the disease [6, 7]. Neuroinflammation plays a significant role in MS pathology, contributing to disease severity and leading to neurodegeneration [8]. Moreover, previous research has linked neuroinflammation to α-synucleinopathies [9]. α-synuclein is a chaperone protein predominantly localized in neuronal synaptic terminals, and its primary physiological function is enhancing synaptic activity [10]. α-synuclein has been implicated in the pathogenesis of Parkinson’s disease and Lewy body dementia. In mouse models, overexpression of α-synuclein has been associated with dysregulation of inflammatory mediators [11]. Prolonged α-synuclein expression has been observed to cause the loss of dopaminergic neurons and oligodendrocytes in the midbrain of marmosets [12]. Additionally, insights from animal models [13–21] have demonstrated α-synuclein’s association with microglial stimulation, axonal damage, and neurodegeneration.

Several animal and human studies have explored the potential role of α-synuclein in MS pathogenesis. In the first animal model study by Papadopoulos et al., α-synuclein overexpression was observed in neurons and glial cells near plaque-containing brain regions [22]. Subsequent studies reported elevated α-synuclein levels in the cerebrospinal fluid (CSF) of MS patients, suggesting its potential as an indicator of axonal injury in demyelinating diseases, including MS [23, 24]. Preliminary analysis of MS tissues also revealed diffuse α-synuclein on plaque edges, suggesting that initial toxic factor expression in neurons could trigger pathological activity in MS [25]. Furthermore, low α-synuclein levels have been linked to rapid deterioration in executive function, memory, and language [26].

In addition, a study in a murine experimental autoimmune encephalomyelitis (EAE) model found that low α-synuclein levels were associated with increased neuroinflammation through a TH-1 cell-mediated immune response [11]. Another study reported that α-synuclein could modulate the differentiation and activation of regulatory T cells (Tregs), a type of immune cell crucial for maintaining immune tolerance and suppressing autoimmune responses [27]. Further research is necessary to clarify α-synuclein’s role in MS pathogenesis and assess its potential clinical use as a biomarker for disease activity and progression. These studies could benefit from using a carefully selected patient population and considering MS as a multifactorial and heterogeneous disease [28, 29].

The primary objective of this study was to identify potential prognostic indicators associated with disease activity in MS. To achieve this, we analyzed the levels of α-synuclein and IL-6 in the peripheral blood of MS patients and compared them with those of a control group while verifying these levels through PBMCs and serum profiling. By employing statistical methods, we explored the relationship between various clinical variables, such as disease duration, Kurtzke’s Expanded Disability Status Scale (EDSS) score [30], and tobacco exposure, and compared them with the expression profiles of both α-synuclein and IL-6. Through these analyses, we aimed to gain insight into the potential utility of α-synuclein as a prognostic biomarker in MS and identify clinical factors associated with disease severity that may influence α-synuclein expression levels.

## 2. Material and methods

### 2.1. Standard protocol approvals and patient consent

Sampling procedures for this study were conducted in accordance with the guidelines of the Ethical Review Committee (ERC-70-2021) and approved by the Institutional Review Board (IRB-307/07-2021-B) of Forman Christian College (a Chartered University) in Lahore, Pakistan. All recruited individuals provided written consent before participating in the study. The study adhered to the principles of the Helsinki Declaration.

### 2.2 Participants recruitment

We prospectively selected 51 MS patients at the Punjab Institute of Neuroscience in Lahore, Pakistan. Samples were collected using strict inclusion and exclusion criteria. Our earlier publication detailed the recruitment process for MS patients and healthy adults [31].

### 2.3 Blood sampling

Whole blood was collected from all participants in vacutainer vials. Serum was obtained by centrifuging the blood at 1300 × g for 10 min. The collected serum samples were transferred to polypropylene microtubes within two h of blood collection and stored at -80°C until further analysis. For RNA extraction, blood samples were promptly analyzed for RNA separation and cDNA synthesis.

### 2.4 Extracting RNA

For RNA extraction from blood, the tube contents were transferred to a 15-ml polypropylene conical centrifuge tube containing 10 ml of RBC lysis buffer (NH_4_Cl, KHCO_3_, and 0.5 M EDTA). Cells were pelleted at 30°C and 1500 rpm. The contents were then transferred to a microcentrifuge tube and centrifuged at 6000 rpm. A Trizol solution (500 μl, MRC, Catalog No. RT111) was added to each tube containing pelleted cells, followed by the addition of 500 μl of chloroform and 0.5 M acetic acid. The solution was centrifuged at 15,000 rpm for 15 min at 4°C. The upper phase was transferred to a separate clean microfuge tube. An equal volume of absolute isopropanol was added and chilled at -20°C to precipitate. Samples were centrifuged at 13,000 rpm for 10 min at 4°C. The clear pellets obtained were washed twice with 70% ethanol. Finally, pellets were air-dried and resuspended in RNase-free water.

### 2.5 Extracted RNA quality assessment, cDNA synthesis, and qRT-PCR analysis

Extracted RNA was quantified using spectrophotometry (NanoDrop Thermo Scientific 2000c). The quality of RNA was analyzed using a 0.7% agarose gel, and gel bands were visualized with an imager (Gel-Doc-It 310 Imager). cDNA was synthesized from total RNA using the Thermo Fisher Scientific RevertAid First Strand cDNA Synthesis Kit (Catalog No. K1622). Before qRT-PCR, conditions for each gene were optimized on a gradient thermocycler (Applied Biosystems). Quantitative analysis was performed using a CFX96 Real-Time PCR system (Bio-Rad) according to the manufacturer’s instructions. The reaction mixture was provided in the qRT-PCR kit for SYBR Green I (Thermo Scientific, Catalog No. K0221). At the end of each run, a melting curve analysis was performed to determine the specificity of the primers. Gene expression levels were normalized to the reference genes using the relative quantification method [32].

### 2.6 Enzyme-linked immunosorbent assay

Serum α-synuclein and IL-6 levels were evaluated according to the manufacturer’s instructions (Zokyo ELISA kits, Wuhan, China). Approximately 50 μL of a standard solution, a blank, and a diluted sample of interest were added to the appropriate wells and incubated for 2 h at 37°C. The conjugate reagent was then added and incubated for 1 h at 37°C. Plates were washed three times, and 50 μL of detection reagent and substrate solution were added before incubating the plates at room temperature for 30 min in the dark. Finally, 50 μL of a stop solution was added, and the plates were immediately analyzed using a microplate reader at an absorbance of 450 nm.

### 2.7 Statistical analysis

GraphPad Prism V9.0 (La Jolla, CA, USA) was used for statistical analyses. To visualize significant fold changes in target gene expression (α-synuclein and IL-6), ΔΔCt was quantified in real-time, followed by the log_2_ fold change. The Mann-Whitney U test was used to analyze the levels of α-synuclein and IL-6 between the patient and healthy groups. Principal component analysis was subsequently applied to the data. The correlation between expression data and various clinical variables was analyzed by Spearman’s or Pearson’s rank correlation, depending on the data distribution. Receiver operating characteristic (ROC) curves were derived from logistic regression to investigate the discriminatory power of selected genes among the disease group and control group. All statistical tests were two-tailed, and a p-value of 0.05 was the threshold for significance.

## 3. Results

### 3.1 Patient characteristics

The patients’ clinical characteristics have been described in our previous publication [31].

### 3.2 Expression levels of α-synuclein and IL-6 in recruited individuals

**Fig 1:**
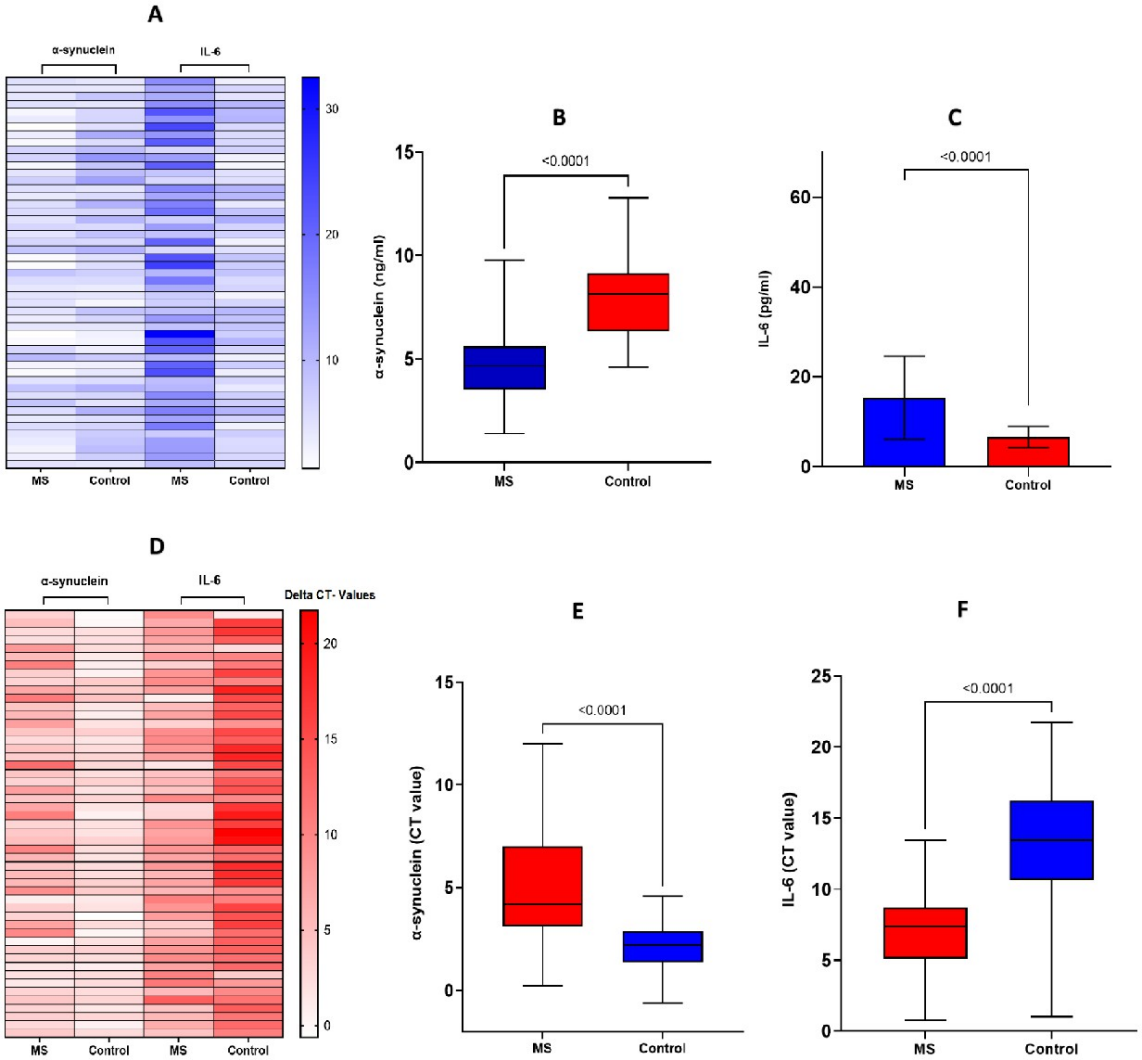
Differential expression of α-synuclein and IL-6 in MS patients compared to the control group. **(A)** Differential gene expression is graphically illustrated with the help of a heatmap. Values representing α-synuclein levels and IL-6 are in ng/ml and pg/ml. Blue indicates higher expression and white indicates lower expression. **(B)** serum levels of α-synuclein and **(C)** IL-6 were significantly increased in MS patients when compared to controls *(p*<0.0001). **(D)** A heat map of CT values is shown between MS and the control group. Red is indicating a lower expression and white is denoting a higher expression. Delta CT-values of α-synuclein **(E)** and IL-6 **(F)** levels are compared between MS and the control group.

The graphical representation of α-synuclein expression in the MS group and control is illustrated in Fig 1(a)–1(f). The transcription profiles of recruited patients revealed that α-synuclein levels are significantly downregulated in MS patients, while IL-6 levels were significantly upregulated. Results of serum quantification demonstrated a similarly distinctive pattern of serum α-synuclein levels in MS patients (4.62 ± 2.00 ng/ml) (mean ± SD) compared to the control group (7.94 ± 2.08 ng/ml). Moreover, the average IL-6 levels were significantly higher in the MS group (15.35 ± 9.25 pg/ml) than in the control group (6.625 ± 2.37 pg/ml).

### 3.3 Distinctive profiles of α-synuclein and IL-6 in the MS and control groups

**Fig 2:**
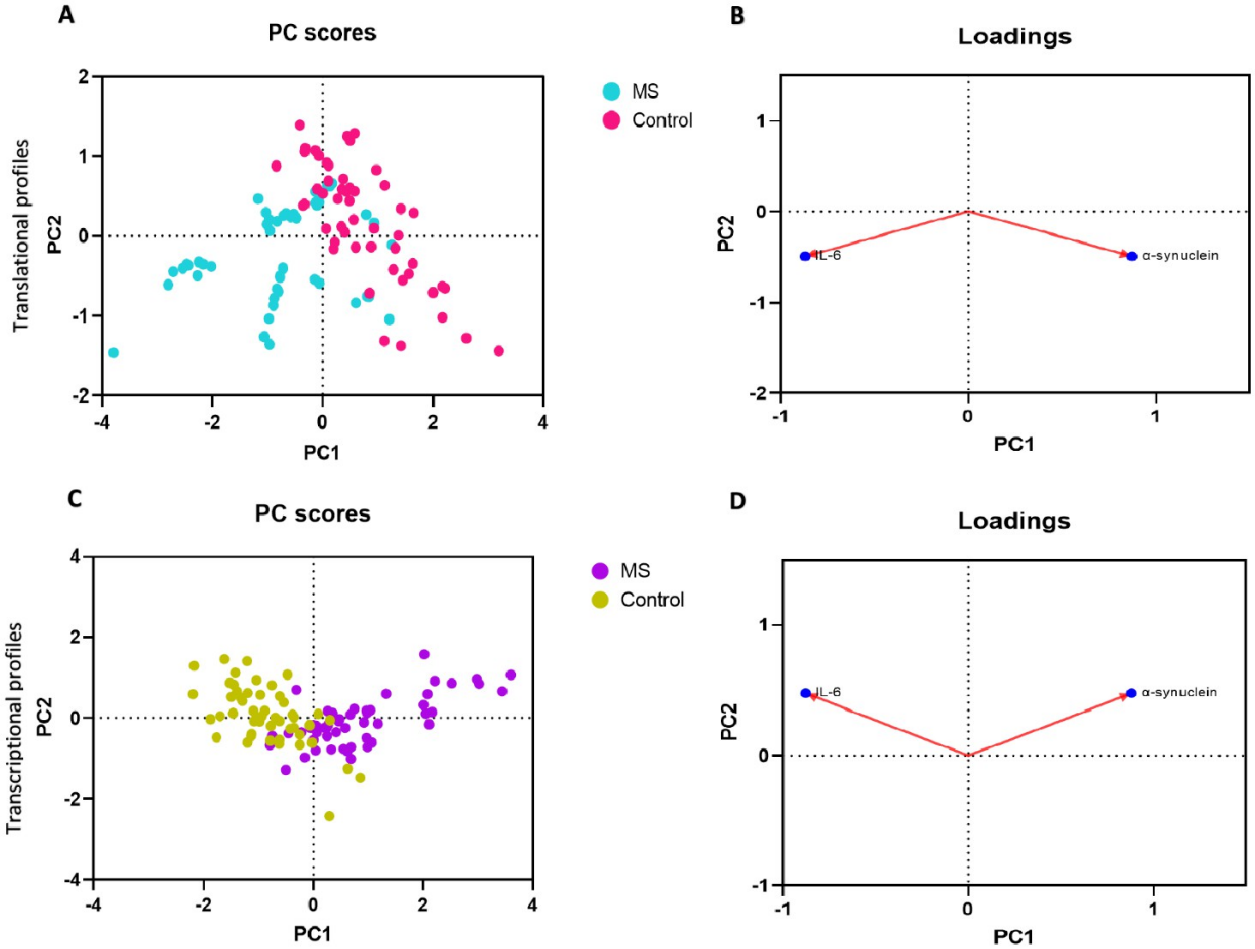
PCA was performed on PBMCs and serum data. PBMC profiles obtained are shown in **(A)** and **(B)**. Serum data are shown in **(C)** and **(D)**. Individual plots are illustrated in panels **(A)** and **(C)**. Samples are represented by dots and each color represents a group of individuals who participated. Panel **(B)** and **(D)** are the variable plots. Arrow graphically represents the direction of each gene. The longer the arrow, the more it influences the variance.

We employed principal component analysis (PCA) to obtain an overview of gene expression of α-synuclein and IL-6 in patients diagnosed with MS in comparison to the control group. The individual plot exhibited statistical clustering between the MS and control groups. The results suggest that the PCA effectively separated the recruited individuals into their respective groups (Fig 2).

### 3.4 Strong discriminatory power of α-synuclein as a diagnostic biomarker

**Fig 3:**
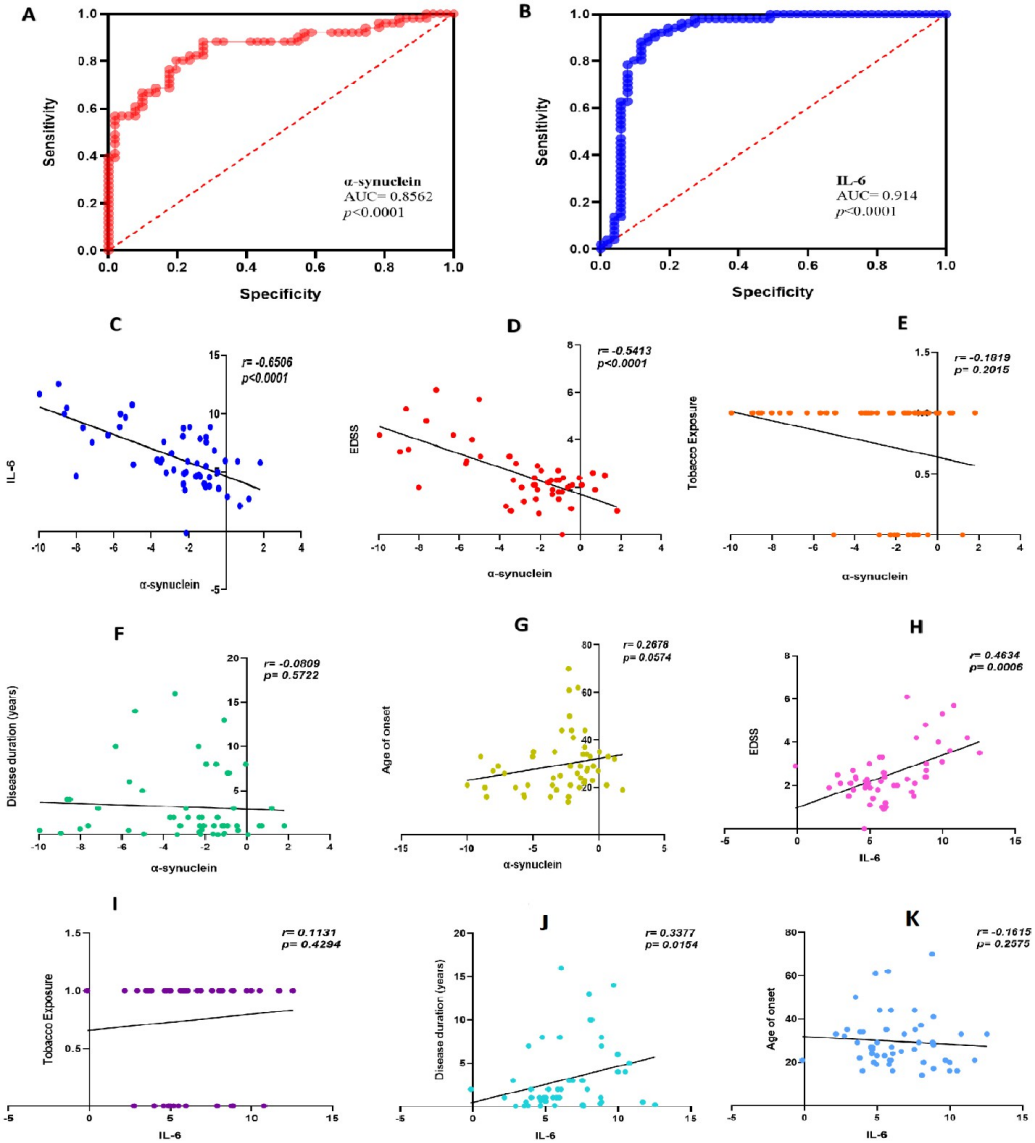
The PBMCs discriminatory profile of α-synuclein correlates with EDSS and predicts disease severity. **(A)** PBMC profiles of α-synuclein and **(B)** IL-6 have AUC values of 0.856 (95% confidence interval 0.781–0.931) and 0.914 (95% confidence interval of 0.849–0.979), with the standard errors of 0.038 and 0.030, respectively. The PBMCs’ levels of α-synuclein were shown to be linked with IL-6 **(C)** age of onset EDSS (**D)**, tobacco exposure **(E)**, disease duration (**F)**, and age of onset **(G)**. In addition, IL-6 was associated with EDSS **(H)**, tobacco exposure **(I)**, disease duration (**J)**, and age of onset **(K)**. The R-value and p-value of α-synuclein and IL-6 were calculated using the person rank correlation coefficient, whereas clinical variables and gene expression were determined using Spearman’s rank correlation.

**Fig 4:**
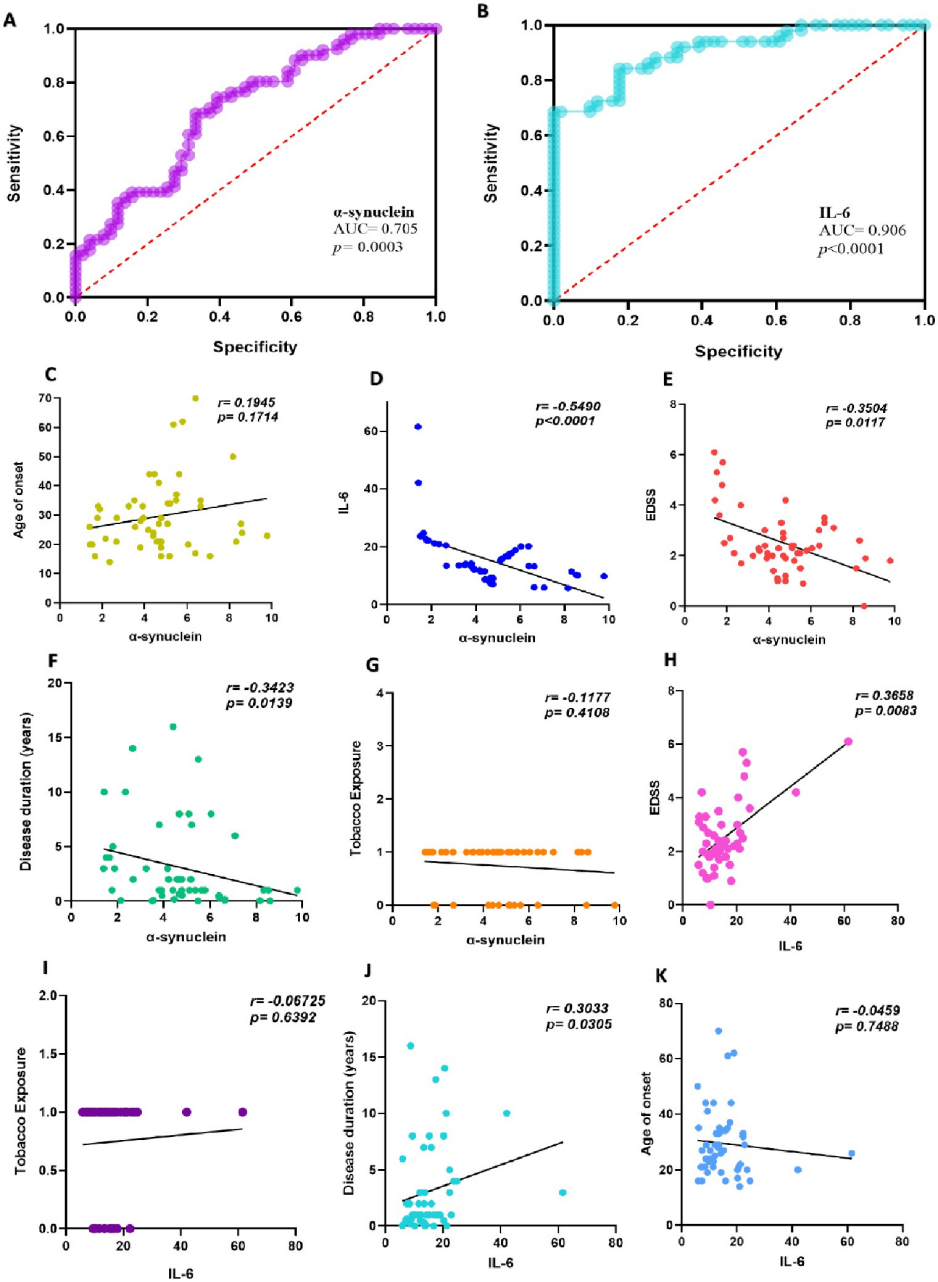
Serum discriminatory profile of α-synuclein also correlates with EDSS and predicts disease severity. **(A)** The discriminatory power of α-synuclein and (**B)** IL-6 serum profiles returned area under the curve (AUC) values of 0.705 (Std.error = 0.050 and 95% confidence interval 0.605 to 0.805) and 0.906 (Std.error = 0.028 and 95% confidence interval 0.850 to 0.962), respectively. Serum levels of α-synuclein were associated with the age of onset **(C)**, IL-6 **(D)**, EDSS (**E)**, disease duration (**F)**, and tobacco exposure **(G)**. In addition, IL-6 was correlated with tobacco exposure **(H)**, EDSS (**I)**, disease duration (**J)**, and age of onset **(K)**. The R-value and p-value of α-synuclein and IL-6 were calculated using the Pearsons’ rank correlation coefficient, whereas clinical variables and gene expression were determined using Spearman’s rank correlation.

Receiver operating characteristic (ROC) analysis was employed to evaluate the test’s ability to distinguish between the MS group and the control group. The test’s quality was measured by the AUC, with an acceptable range of 0.7–0.8, an excellent range of 0.8–0.9, and an outstanding range above 0.9. We present two logistic regression models using our patients’ PBMC (Fig 3(a) and 3(b)) and serum (Fig 4(a) and 4(b)) profiles. The performance of both models was estimated, demonstrating a strong discriminatory factor. These results indicate that the expression of IL-6 and α-synuclein serve as excellent predictors of MS.

### 3.5 The PBMCs and serum levels of α-synuclein are significantly correlated with the magnitude of MS disease severity

The PBMCs and serum profiles of α-synuclein demonstrated a significant negative correlation with EDSS (r = -0.541, p < 0.0001) and (r = -0.350, p = 0.0117), indicating that α-synuclein may serve as a potential prognostic biomarker for MS. A strong negative correlation was observed between α-synuclein and IL-6 in both PBMCs data (r = -0.650, p < 0.0001) and serum data (r = -0.549, p < 0.0001). The age of onset and α-synuclein PBMCs (r = 0.2678, p = 0.0574) and serum profiles (r = 0.1945, p = 0.1714) were not significantly correlated. Additionally, α-synuclein showed no significant correlation with tobacco exposure for both the PBMC (r = -0.1819, p = 0.2015) and serum (r = -0.1177, p = 0.4108) levels. Notably, the disease duration of MS patients was significantly correlated with the serum profile (r = -0.3423, p = 0.0139) but not with the PBMCs profile of α-synuclein (r = -0.0809, p = 0.5722).

We also investigated the relationship between the PBMCs and serum levels of IL-6 with clinical parameters. A significant correlation was found between EDSS and IL-6 PBMCs levels (r = 0.4634, p = 0.006) and serum levels (r = 0.3658, p = 0.0083). Additionally, the disease duration of the recruited patients was significantly correlated with PBMCs levels (r = 0.3377, p = 0.0154); however, no significant correlation was found with serum IL-6 profiles (r = 0.3033, p = 0.3045). IL-6 also showed no significant correlation with tobacco exposure and age of disease onset. Graphs illustrating the correlational analysis are provided in Figs 3(c)–3(k) and 4(c)–(4k).

## Discussion

Chronic inflammation is the hallmark of neurodegenerative diseases. MS is an autoimmune disorder of the central nervous system and is now recognized as a neurodegenerative disease [33]. This autoimmune disease affects the central nervous system and is characterized by axonal and neuronal loss [8]. Neurodegeneration and neuroinflammation are also associated with α-synucleinopathies, a class of diseases related to the aggregation and accumulation of α-synuclein protein [34]. Although α-synuclein is primarily an intracellular protein, it can also be found in other biological fluids such as CSF and blood [35, 36]. α-synuclein is expressed in various regions of the brain, including the substantia nigra, thalamus, cortex, and hippocampus, where it regulates various cellular processes, including inflammation and release of neurotransmitters and cytokines [37–40].

The role of α-synuclein in MS pathology is an area that requires further investigation [22]. While some animal model studies have found increased expression of α-synuclein in MS lesions and surrounding neurons and glial cells, suggesting a potential link between α-synuclein and neuroinflammation [41, 42]. Immunoreactivity for α-synuclein has also been detected in MS lesions with enhanced inflammatory activity, further supporting this association [42, 43]. Studies have reported higher levels of α-synuclein in the CSF of MS patients, and these levels are positively correlated with disease severity and neuroinflammation [42–45]. In contrast, α-synuclein levels were found to be downregulated in the CSF of patients with CIS, a precursor to MS, when compared to a control group [45]. Furthermore, the study found that the levels of α-synuclein in the CSF of patients with CIS were negatively correlated with the number of T2-weighted magnetic resonance imaging (MRI) lesions in MS patients [45]. This suggests that lower levels of α-synuclein in the CSF may be associated with increased MS-related brain damage.

IL-6 mediates various biological processes, including the regulation of immune reactions. During the adaptive immune response, it promotes the differentiation of naive CD4+ T cells into T helper 17 cells and negatively regulates TGF-β-induced regulatory T cell (Treg) differentiation [46–48]. Dysregulation of IL-6 leads to an imbalance between Th17 and Treg cells, resulting in the development of autoimmune disorders [49]. Therefore, systemic lupus, rheumatoid arthritis, epileptogenesis, and MS patients have been found to exhibit enhanced inflammation, likely due to dysregulated IL-6 activity [50–52]. IL-6 overproduction has been linked to a number of autoimmune disorders, including MS [53]. Increased levels of IL-6 have been found in MS patients’ blood, CSF, and nervous tissue [50]. Moreover, animal models have shown that IL-6-deficient mice exhibit resistance to the development of experimental autoimmune encephalomyelitis (EAE) symptoms [54, 55]. These implications suggest IL-6’s pathological role in MS, which further emphasizes the usefulness of IL-6 inhibitor therapy in the possible prevention of relapses of MS.

In this study, we observed high expression of IL-6 in both PBMCs and serum and found a significant correlation with EDSS and disease duration. Similarly, increased levels of IL-6 have been reported and correlated with disease duration [50]. Enhanced IL-6 levels were also reported in the monocytes of MS patients [56]. Previously, small-group studies have correlated serum and CSF IL-6 levels with EDSS scores [57, 58]. Another study investigated the levels of IL-6 in all MS phenotypes. They found that dysregulated IL-6 levels were associated with MS, regardless of its phenotypes. Furthermore, IL-6 levels were found to be associated with disease activity and the number of relapses in the previous year [59]. These findings suggest that IL-6 levels can serve as a marker of disease activity and disability in MS patients. Monitoring IL-6 levels may provide clinicians with a useful tool for assessing disease activity and treatment response in MS patients.

In the present investigation, we employed ELISA and qRT-PCR techniques to analyze α-synuclein and IL-6 levels in peripheral blood samples, which are more easily accessible than brain tissue or CSF. We found that MS patients had significantly lower PBMCs and serum α-synuclein levels than the healthy control group. This outcome is consistent with Nuray et al.’s research, which also revealed decreased α-synuclein levels in the serum of MS patients relative to controls [60]. However, Nuray et al. included patients who received cortisone therapy, which may have influenced the accuracy of their findings. Additionally, they discovered no significant correlation between α-synuclein and EDSS or disease duration. In contrast, our study found a significant relationship between serum and PBMCs α-synuclein levels with EDSS, indicating that lower α-synuclein levels may be linked to disease progression. Nevertheless, we did not find any connection between α-synuclein serum levels and disease duration, which agrees with Nuray et al.’s results [60]. This suggests that α-synuclein could be a promising biomarker for MS disease severity. However, further studies are necessary to validate this association and investigate the underlying mechanisms.

This study investigated the PBMCs and serum profiles of α-synuclein and IL-6 in patients with MS and a control group. The results showed that α-synuclein levels were significantly downregulated, while IL-6 levels were significantly upregulated in MS patients compared to controls. The PBMCs and serum profiles of α-synuclein showed a significant negative correlation with the Expanded Disability Status Scale (EDSS) and disease severity. The study also found a codependency between α-synuclein and IL-6. The PBMCs and serum levels of IL-6 were significantly correlated with EDSS, while the disease duration of MS patients was significantly correlated with IL-6 PBMCs levels. However, one potential limitation of our study is that disease severity was assessed without a time component, and we only used one source of fluid biomarker for biomarker development. Therefore, longitudinal studies with multiple sources of biomarkers are needed for more robust evaluations. Moreover, we only investigated the association between IL-6 and α-synuclein levels in MS patients, but other inflammatory cytokines such as IL-1β, IL-10, and TNF-α should be explored in future studies.

## Conclusions

Our study provides evidence that MS patients exhibit significantly lower levels of α-synuclein and higher levels of IL-6 in peripheral blood compared to healthy controls. Furthermore, we discovered a significant correlation between α-synuclein levels and disease severity measures in MS patients, suggesting that α-synuclein may function as a potential prognostic biomarker. However, the diagnostic and prognostic utility of α-synuclein in MS patients necessitates further investigation in larger cohorts and additional biofluids, such as CSF. Additionally, the relationship between α-synuclein levels and neurocognitive outcomes in MS patients should be explored. Our findings contribute to the development of peripheral biomarkers for MS diagnosis and prognosis, which can assist in managing this complex neurological disorder.

## Supporting information

S1 Data (XLSX)
